# Identification and characterization of Toll-like receptor 14d in Northeast Chinese lamprey (*Lethenteron morii*)

**DOI:** 10.3389/fimmu.2023.1153628

**Published:** 2023-04-18

**Authors:** Zebin Zhou, Shaoqing Ding, Yaqian Wang, Janfeng Ren, Xiangyang Zhang, Weiming Li, Qinghua Zhang

**Affiliations:** ^1^ Key Laboratory of Exploration and Utilization of Aquatic Genetic Resources, Ministry of Education, Shanghai Ocean University, Shanghai, China; ^2^ International Research Center for Marine Biosciences, Ministry of Science and Technology, Shanghai Ocean University, Shanghai, China; ^3^ Key Laboratory of Marine Biotechnology of Fujian Province, Institute of Oceanology, Fujian Agriculture and Forestry University, Fuzhou, China; ^4^ Department of Fisheries and Wildlife, Michigan State University, East Lansing, MI, United States

**Keywords:** innate immunity, Lamprey, Toll-like receptor, adaptor molecule, NF-kappaB, MyD88, TRIF

## Abstract

Toll-like receptors (TLRs) play an important role in innate immunity of defense against bacterial or viral pathogens. To study the biological characteristics and functions of the TLR genes, TLR14d was identified from Northeast Chinese lamprey (*Lethenteron morii*) and named LmTLR14d. LmTLR14d coding sequence (cds) is 3285 bp in length and encodes 1094 amino acids (aa). The results showed that LmTLR14d has the typical structure of TLR molecule, which contains the extracellular domain of leucine-rich repeats (LRR), transmembrane domain, and intracellular domain of Toll/interleukin-1 receptor (TIR). The phylogenetic tree showed that LmTLR14d is a homologous gene of TLR14/18 in bony fish. Quantitative real-time PCR (qPCR) revealed that LmTLR14d was expressed in various healthy tissues, including immune and non-immune tissues. *Pseudomonas aeruginosa* infection up-regulated LmTLR14d in the supraneural body (SB), gill, and kidney tissues of infected Northeast Chinese lamprey. Immunofluorescence results showed that LmTLR14d was located in the cytoplasm of HEK 293T cells in clusters, and its subcellular localization was determined by the TIR domain. The immunoprecipitation results showed that LmTLR14d could recruit *L.morii* MyD88 (LmMyD88) but not *L.morii* TRIF (LmTRIF). Dual luciferase reporter results showed that LmTLR14d significantly enhanced the activity of *L.morii* NF-κβ (LmNF-κβ) promoter. Furthermore, co-transfection of LmTLR14d with MyD88 significantly enhanced the *L.morii* NF-κβ (LmNF-κβ) promoter activity. LmTLR14d can induce the expression of inflammatory cytokine genes *il-6* and *tnf-α* downstream of NF-κB signal. This study suggested that LmTLR14d might play an important role in the innate immune signal transduction process of lamprey and revealed the origin and function of teleost-specific TLR14.

## Introduction

Mammals and birds mainly rely on their adaptive immunity to defend against bacterial or viral pathogens, while fish and amphibians as well as insects mainly rely on the innate immune system (1, 2). The innate immune system largely relies on pattern-recognition receptors (PRRs) expressed on the cell surface to recognize pathogen-associated molecular patterns (PAMPs). Toll-like receptors (TLRs), which constitute an important PRR family, recognize conservative PAMPs and trigger signaling pathways that regulate the innate immune system (3).

Members of TLR family are type I transmembrane proteins that are characterized by an N-terminus with variable numbers of extracellular leucine-rich repeat (LRR) domains, a transmembrane domain, and a C-terminal intracellular toll/interleukin-1 receptor (TIR) homology domain. The extracellular domain is composed of repetitive LRR tandem sequences and is responsible for recognizing PAMP (4). The transmembrane region and the intracellular TIR domain are highly conserved (5, 6). There is a region with three conservative boxes (box1, box2, and box3) in the TIR domain, which are the core components for regulating downstream signal transduction (7, 8).

Thirteen TLRs have been found in mammals, including 10 TLRs (TLR1-TLR10) in humans and 13 TLRs (TLR1-TLR13) in mice (9, 10). In addition, unique TLRs have been identified in teleosts due to the additional whole genome duplication in these vertebrate animals (11, 12). Teleost TLRs consist of 6 subfamilies, including TLR1, TLR3, TLR4, TLR5, TLR7, and TLR11. The TLR1 subfamily includes TLR1, TLR2, TLR6, TLR10, and the teleost-specific TLR14, TLR18, TLR25, TLR27, and TLR28. However, TLR6 and TLR10 are not found in teleosts (13). In a series of specific TLRs, TLR14 was first identified in tiger puffer (*Takifugu rubripes*) (14, 15), while TLR18 was first identified in zebrafish (*Danio rerio*) and tiger puffer (16). As the earliest representative species of fish for genome sequencing, the TLR classification of zebrafish and tiger puffer determines the subsequent TLR classification and identification of other fish to some extent. However, at present, fugu TLR14 and fugu TLR18 are the same gene (tiger puffer TLR14 accession number: AC156431.1, tiger puffer TLR18 accession number: LOC101068527; Commonly known as tiger puffer TLR14) that had been originally named differently. Therefore, fish TLR14 and TLR18 are considered as orthologous (10), and they are usually discussed together (17, 18). TLR14 shares some features with TLR1, TLR6, and TLR10, which are members of subfamily TLR1, and plays an important role in the resistance of fish to aquatic pathogens (19). In recent years, TLR14 has been identified in several fish species, such as Japanese flounder (*Paralichthys olivaceus*) (20), miiuy croaker (*Miichthys miiuy*) (21), orange-spotted grouper (*Epinephelus coioides*) (17), golden pompano (*Trachinotus ovatus*) (22), mandarin fish (*Siniperca chuatsi*) (23) and Asian swamp eel (*Monopterus albus*) (24).

Northeast Chinese lamprey (*Lethenteron morii*) is a jawless vertebrate species that belongs to Cyclostomata, Petromyzontiformes, Petromyzontidae, and *Lampetra*. It has no complete thymus, and its lymphoid cells do not differentiate into T lymphocyte and B lymphocyte (25). Lampreys have unique variable lymphocyte receptors (VLRs), which contain arrays of LRRs and may function in soluble as well as membrane-anchored forms, might be derived from TLRs. Thus, lampreys are considered a useful model for studying the evolution of immunity (26). TLRs are thought to epitomize the evolutionary conservation of a biological system (14).

Research on TLR genes of jawless vertebrates is less extensive than those in mammals and teleosts. There are 16 TLRs in the genome assemblies of sea lamprey and Japanese lamprey. Lamprey TLRs are consisted of both fish (F)- and mammalian (M)-type TLRs, with TLR14 differentiated into TLR14a, TLR14b, TLR14c and TLR14d in the sea lamprey (*Petromyzon marinus*) as well as TLR14a and TLR14b in Japanese lamprey (*Lampetra japonica*) (27, 28). The classification of lamprey TLR was based on the human and fugu TLRs (27, 28). With the identification of TLR types in an increasing number of fish species, further homologous analysis of TLR of lamprey has become possible. Recently, Quan et al. identified the TLR3 gene of lamprey and analyzed its tissue expression (29). However, TLRs function and the involved signaling pathways are not yet clear in lamprey. In this study, we aim to examine the structure and function of TLR14d in Northeast Chinese lamprey. Here we report the molecular characteristics as well as constitutive and inducible expression of TLR14d in Northeast Chinese lamprey (LmTLR14d) and its function in the innate immune signal transduction process of the NF-κB pathway. This study will help us fully understand the origin and function of fish specific TLRs in innate immune responses.

## Materials and methods

### Fish and bacterium

The healthy Northeast Chinese lampreys were collected from a tributary of Yalu River in Dandong city, Liaoning Province, China. They were temporarily held at a water temperature of 8 ± 1 °C. The average body length of larvae was 11.5 ± 0.8 cm, and the body weight was 5.2 ± 0.6 g. The average body length of the adults was 30.1 ± 3.2 cm, and the body weight was 36.1 ± 2.1 g. The proposed research methodology received clearance from the Shanghai Ocean University Experimentation Ethics Review Committee (SHOU-DW-2016-003). Lampreys were handled according to the procedures of the Institutional Animal Care and Use Committee of Shanghai Ocean University, Shanghai, China.

*Pseudomonas aeruginosa* PA11 strain was isolated from diseased Northeast Chinese lamprey and stored at -80°C in our laboratory. It was used to challenge the healthy larvae and adult lampreys in the following experiments.

### Extraction and cDNA synthesis

From four healthy larvae and adult Northeast Chinese lampreys nine tissues, including brain, intestine, heart, gill, liver, muscle, kidney, supraneural body (SB), and skin, were collected for this study. Total RNA from Northeast Chinese lamprey and HEK 293T cells were extracted by Trizol method and RNA samples using 1% agarose gel electrophoresis validation and NanoDrop 2000 to assess sample concentration and stored at -80°C. In addition, adult lampreys were injected intraperitoneally with *P. aeruginosa* at a dose of 500 μL/100 g body mass and a concentration of 1.96 x 10^6^ CFU/ml. Total RNA was extracted from SB, gill, intestine and kidney tissues at 0, 6, 12, 24, 48 and 72 h after injection and stored at -80°C. Reverse transcription was performed using PrimeScript RT reagent Kit with gDNA Eraser (Takara Bio, Beijing, China).

### LmTLR14d clone

The preliminary sequence of LmTLR14d was obtained by the BLAST method from the genomes of sea lamprey (https://genomes.stowers.org/organism/Petromyzon/marinus) and Japanese lamprey (http://jlampreygenome.imcb.a-star.edu.sg/blast/). The primers were designed by using highly conserved homologous genes from vertebrates (**Supplementary Table 1**). The primers were synthesized by Sangon Biotech Co., Ltd. (Shanghai, China). PCR amplification was performed using mixed cDNA of healthy tissue from Northeast Chinese lamprey as a template. The recovered products were inserted into pMD-19T plasmid (Takara Bio, Beijing, China) and the positive clones were sequenced (Sangon Biotech Co., Ltd.; Shanghai, China).

### LmTLR14d sequence analysis

The BLAST tool in NCBI (http://www.ncbi.nlm.nih.gov/BLAST) was used to identify homologous sequences of LmTLR14d. The open reading frame (ORF) was deduced by Getorf software (http://emboss.bioinformatics.nl/cgi-bin/emboss/getorf). The protein molecular weight and isoelectric point were deduced by ExPASy software (http://web.expasy.org/protparam/). The protein domain was deduced and analyzed by SMART software (http://smart.embl.de/) and PROSITE software (https://prosite.expasy.org/). DNAMAN 6.0 and ClustalX 2.0 were used for amino acid sequence alignment, Neighbor-joining method in MEGA X and iTOL software (http://itol.embl.de/) were used to construct a phylogenetic tree. The LmTLR14d extracellular domain (ECD) structural model was constructed using SWISS (https://swissmodel.expasy.org/).

### Quantitative real-time PCR analysis

Quantitative real-time PCR (qPCR) analysis was used to detect the *TLR14d* expression in different tissues of Northeast Chinese lamprey and the effect of LmTLR14d overexpression on inflammatory cytokines in HEK 293T cells. *β-actin* was used as internal reference gene *in vivo* and *EF-1* as internal reference gene *in vitro.* The primers used for qPCR are detailed (**Supplementary Table 1**). *LmTLR14d* of the SB, gill, intestine, and kidney from healthy and infected by *P. aeruginosa* were detected at different time points to analyze the relative expression levels. The reaction conditions for PCR were 50°C for 2 min, 95°C for 10 min; 95°C for 10 s, 60°C for 30 s, amplification for 40 cycles; 95°C for 30 s, 60°C for 30 s, 40°C for 30 s. Each reaction was carried out in triplicates and a melting curve analysis was performed to confirm the specificity of the reactions. The average ΔCT value was calculated by subtracting the average *β-actin* CT from the average target gene CT. The -ΔΔCT was calculated by subtracting the control ΔCT from the treatment ΔCT. The relative quantity of mRNA was calculated as 2^-(ΔΔCT)^.

### Cellular immunofluorescence

The expression plasmid pCMV-Tag 2B was used to amplify the target fragment with primers containing restriction sites. pCMV-LmTLR14d, pCMV-LmTLR14d ΔLRR and pCMV-LmTLR14d ΔTIR were digested with *Bam*HI and *Eco*RI (**Supplementary Table 1**) (**Supplementary Figure 1**). pCMV-HEK 293T cells were inoculated in a 35 mm glass dish (NEST). The pCMV-LmTLR14d, pCMV-LmTLR14d ΔLRR, pCMV-LmTLR14d ΔTIR and pCMV-Tag 2B plasmids were transfected into cells using Fugene HD (Promega) transfection reagent. Cell samples were treated 36 h after transfection. A murine anti-FLAG monoclonal antibody (Sigma) was used as the primary antibody and a goat anti-murine Alexa Fluor 488 (Invitrogen) containing a fluorescein-coupled molecule was used as the secondary antibody. DAPI was used to label the nuclei. The samples were observed and photographed under a confocal microscope.

### Co-immunoprecipiation and Western blotting

Our laboratory has previously identified *L. morii* MyD88 (LmMyD88, accession number MN368861) (30, 31) and *L. morii* TRIF (LmTRIF, accession number OL944500) (30, 31), respectively. Their cds sequences were linked to the eukaryotic expression vector pcDNA3.1(+)-Myc (**Supplementary Figure 2**).

600 ng of pCMV-LmTLR14d or pCMV-Tag 2B were co-transfected with 600 ng of pcDNA3.1(+)-Myc-LmMyD88 or pcDNA3.1(+)-Myc-LmTRIF into 6-well plates of HEK 293T cells (5×10^5^ cells/well). After transfection for 48 h, the cells were lysed with cell lysate (Yeason) added with PMSF at 4°C for 30 min. 50 μL Protein A magnetic beads (Invitrogen) were incubated with murine anti-FLAG monoclonal antibody (Sigma) at 4°C for 2 h. The supernatant after cell lysis (900 μg protein/500 μL sample) was incubated with 50 μL magnetic beads bound with FLAG antibody at 4°C overnight. After the incubation, all test tubes were placed on the chemical magnetic bracket (Cell signaling technology), the supernatant was taken out, and washed with wash buffer three times. Finally, the beads were resuspended in SDS buffer (Takara) and denatured at 95°C for 5 min. The samples were temporarily stored at -20°C for the next step of protein blot detection.

The input (20 μg) and immunoprecipitated (IP) samples (80 μg) were subjected to electrophoresis on SDS-PAGE and transferred to PVDF membrane (Bio-rad). The samples were blocked with PBST buffer containing 5% skimmed milk powder at room temperature for 1 h. The blocking solution was discarded and the mixture containing murine anti-FLAG M2 antibody (1: 3000; Sigma) or murine anti-Myc antibody (1: 3000; Yeason) at 4°C overnight. The membrane was washed with PBST buffer for three times, and HRP-labeled goat anti-mouse antibody (H+L) (1: 3000; Yeason) was added and incubated for 3 h at room temperature. After wash for three times in PBST buffer, chemiluminescence was detected using the ECL developing solution (Bio-rad) in the Amersham Imager 680.

### Luciferase analysis

The expression plasmid pCMV-Tag 2B was used to amplify the target fragment with primers containing restriction sites. The ORF of LmTLR14d was digested with *Bam*HI and *Eco*RI (**Supplementary Table 1**). The sequence of expression plasmid was identified by Sangon Biotech Co., Ltd. (Shanghai, China) for sequencing. The reporter gene of LmNF-κB promoter (accession number MN368861) was linked to reporter vector pGL3-Enhancer plasmid. LmMyD88 and LmTRIF were cloned into pCMV-Tag 2B plasmid, respectively. HEK293T cells were seeded in 24 well plates at 8×10^4^ cells per well for 24 h to transfect. The pCMV-LmTLR14d, pCMV-LmTLR14d LRR, pCMV-LmTLR14d TIR, pCMV-MyD88, pCMV-LmTRIF and NF-κB reporter gene of *L. morii* were co-transfected into cells in different groups with Fugene HD transfection reagent, and phRL-TK plasmid was used as the internal reference. After 36 h of transfection, the activities of firefly luciferase and *Renilla* luciferase were detected using a double luciferase reporter gene detection kit (Promega), and the biology was repeated three times.

### Enzyme linked immunosorbent assay

1000 ng pCMV-LmTLR14d plasmid was transfected into HEK 293T cells cultured in 6-well plates, and pCMV-Tag 2B was used as control. 24 h after transfection, the cells were treated with 30 μg/mL LPS (Beyotime) and 50 μg/mL poly I:C (Sigma) for 12 h. After collecting the supernatant of cell culture, the secretion level of corresponding protein was detected by IL-6 and TNF-α ELISA kit (Jianglaibio). The expression of inflammatory cytokine mRNA was detected by the qPCR methods mentioned above.

### Statistical analysis

All data were analyzed with GraphPad Prism 8.0 software. Bars show mean ± SEM. A one-way ANOVA analysis of variance followed by Bonferroni multiple comparison tests was used to determine the differences among the groups. *P* < 0.05 was accepted significant.

## Results

### Homology and structural characteristic of LmTLR14d

**Figure 1:**
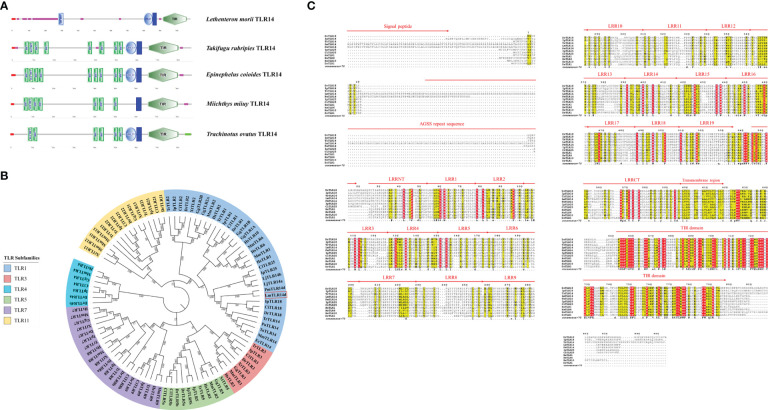
Preliminary domain analysis and homologous relationship of LmTLR14d. **(A)** TLR14 domain features predicted by SMART online software. **(B)** Phylogenetic tree constructed based on LmTLR14d amino acid with Neighbor-joining method. A bootstrap analysis is performed using 1000 replicates to test the relative support for particular clades. The remaining settings use default parameters. The GenBank accession numbers of these sequences are listed in **Supplementary Table 2**. **(C)** Multiple sequence alignment of *L. morii* TLR14d with other species TLR14d. Among them, *Petromyzon marinus* TLR14d (PmTLR14d) was the result of transcript prediction in the NCBI Genebank (XM_032975775.1).

Full length cds of LmTLR14d was cloned from Northeast Chinese lamprey. LmTLR14d (NCBI accession number: OP603966) cds was 3285 bp, encoding 1094 amino acid residues (aa) with a relative molecular weight of 116.09 kD and theoretical isoelectric point of 7.69. The preliminary SMART domain analysis results show that LmTLR14d has TLR characteristics (**Figure 1A**).

A phylogenetic tree of the TLRs of different species was constructed. To enrich the homology database, we found the homologous gene of LmTLR14d in the online genome of sea lamprey by BLAST, which was named PmTLR2 (accession number: XM_032975775.1). To avoid ambiguity, PmTLR2 was called PmTLR14d in this manuscript. The results showed that TLR consisted of 6 subfamilies. TLR1 family was divided into three branches. TLR1, TLR6, and TLR10 clustered into one branch, while fish-specific TLR14, TLR18, and TLR25 clustered into one branch, with TLR2 clustered into the third branch. LjTLR14a and LjTLR14b are closer to fish TLR25 while LmTLR14d is closer to bony fish TLR14/18 (**Figure 1B**). Further multi-sequence alignment results showed that LmTLR14d contained one signal peptide, one repeat sequence encoded by an AGSS repeat sequence, one LRRNT domain, 19 LRR domians, one LRRCT domain, one transmembrane domain, and one TIR domain (**Figure 1C**).

**Figure 2:**
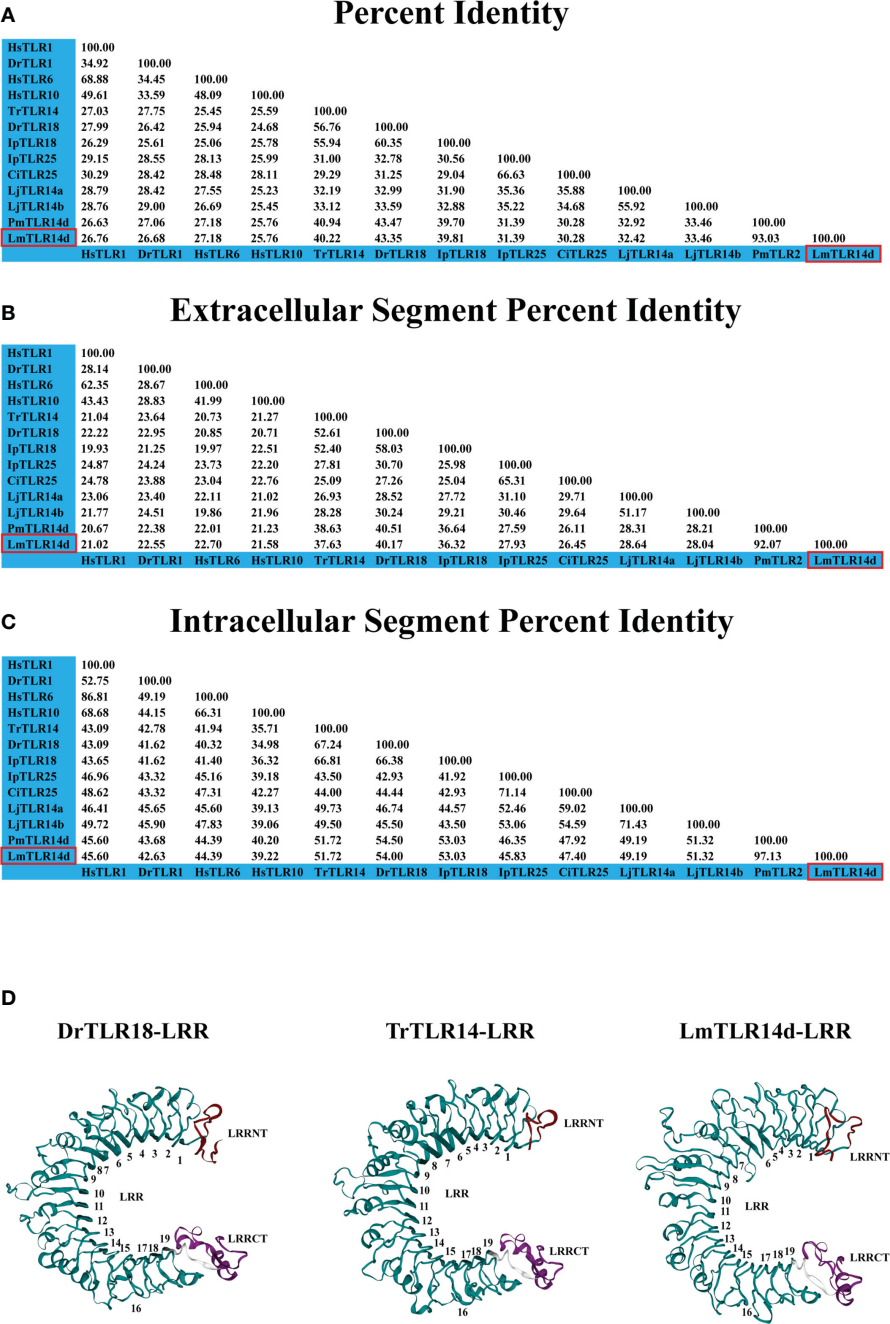
Deep analysis of homology and domain characteristics of LmTLR14d. Sequence full length percent identity **(A)**, extracellular segment percent identity **(B)** intracellular segment percent identity **(C)** analysis of *L. morii* TLR14d. **(D)** The 3D structure of the LmTLR14d, DrTLR18 (*Danio rerio* TLR18) and TrTLR14 (*Takifugu rubripies* TLR14) LRR domain. LRRNT, marked in red; CT, LRRCT, marked in purple; and 1-19, LRR1-LRR19, marked in green.

The comparison of the identity of the TLR1 subfamily between LmTLR14d and vertebrates that have been identified revealed that the aa sequence of LmTLR14d is 26.76-93.03% identical to those of TLRs from other species. It has the highest identity with PmTLR14d (93.03%), the homologous gene of LmTLR14d obtained by BLAST in sea lamprey genome. Except for PmTLR14d, it has the highest identity with zebrafish TLR18 (43.35%). The identity between LmTLR14d and bony fish TLR14/18 (39.81-43.35%) is higher than that between LmTLR14d and Japanese lamprey TLR14a/b (32.42-33.46%). Similar results were also presented in the segmental homology analysis of LmTLR14d and TLR from other species. The results of segmented identity analysis showed that LmTLR14d had the highest identity with the extracellular segment and intracellular segment of zebrafish TLR18 (extracellular segment identity: 40.17%; intracellular segment identity: 54.00%) except PmTLR14d. The identity of extracellular segment and intracellular segment of LmTLR14d and bony fish TLR14/18 (extracellular segment identity: 36.32-40.17%; intracellular segment identity: 51.72-54.00%) is higher than that of LmTLR14d and Japanese lamprey TLR14a/b (extracellular segment identity: 28.04-28.64%; intracellular segment identity: 49.19-51.32%). (**Figures 2A-C**; **Table 1**).

**Table 1:** Brief analysis of LmTLR14d identity.

Alignment sequence	The fish TLR with the highest identity	Identity range with fish TLR	Identity with LjTLR14a	Identity with LjTLR14b
Coding Region Sequence	DrTLR18 (43.35%)	30.28-43.35%	32.42%	33.46%
Extracellular Segment	DrTLR18 (40.17%)	36.32-40.17%	28.64%	28.04%
Intracellular Segment	DrTLR18 (54.00%)	51.72-54.00%	49.19%	51.32%

Compared with the sequences identified from the PDB database, the LmTLR14d ECD had the highest consistency with mouse (*Mus musculus*) TLR2 (PDB ID: 5D3I). Mouse TLR2 was used as the template for homologous modeling, and the constructed model showed that LmTLR14d contained one LRR-N-terminal (LRRNT) domain, 19 LRR domains, and one LRR-C-terminal (LRRCT) domain, which was consistent with the result of multiple sequence alignment (**Figure 2D**). Three conservative box regions were identified in the TIR domain of LmTLR14d, box 1 (-AF-SY-), box 2 (LC-RD-PG) and box 3 (FW-) (**Table 2**). There is a non-conservative site where leucine in box 2 of LmTLR14d becomes cystine.

**Table 2:** Multiple sequence alignments of LmTLR14d TIR domains with those of other vertebrates.

Species TLR	box1	box2	box3
*Homo sapiens* TLR1	FH**AF**I**SY**SG	**IC**LHE**RN**FV**PG**	**FW**ANLR
*Danio rerio* TLR1	FH**AF**V**SY**SQ	**MC**HHE**RD**FI**PG**	**FW**ANLR
*Homo sapiens* TLR6	FH**AF**I**SY**SE	**IC**LHE**RN**FV**PG**	**FW**ANIR
*Homo sapiens* TLR10	FH**AF**I**SY**SE	**IC**LYE**SY**FD**PG**	**FW**ANLR
*Takifugu rubripes* TLR14	YH**AF**I**SY**SH	**LC**IHE**RD**FT**PG**	**FW**AQLR
*Ictalurus punctatus* TLR25	YH**AF**I**SY**SH	**IC**IHE**RD**FV**PG**	**FW**SSLR
*Ctenopharyngodon idella* TLR25	YH**AF**I**SY**SQ	**LC**IHE**RD**FE**PG**	**FW**CNLR
*Danio rerio* TLR18	FH**AF**I**SY**SH	**LC**IHE**RD**FI**PG**	**FW**IQLR
*Ictalurus punctatus* TLR18	FH**AF**I**SY**SH	**LC**IHE**RD**FM**PG**	**FW**EQLR
*Lethenteron morii* TLR14d	FH**AF**I**SY**SH	**VC**VHE**RD**FT**PG**	**FW**AQLR

Three typical boxes in TIR domain, named Box1, Box2 and Box3, are conserved. The GenBank accession numbers of these sequences are listed in **Supplementary Table 2**.

### Expression profile of LmTLR14d

**Figure 3:**
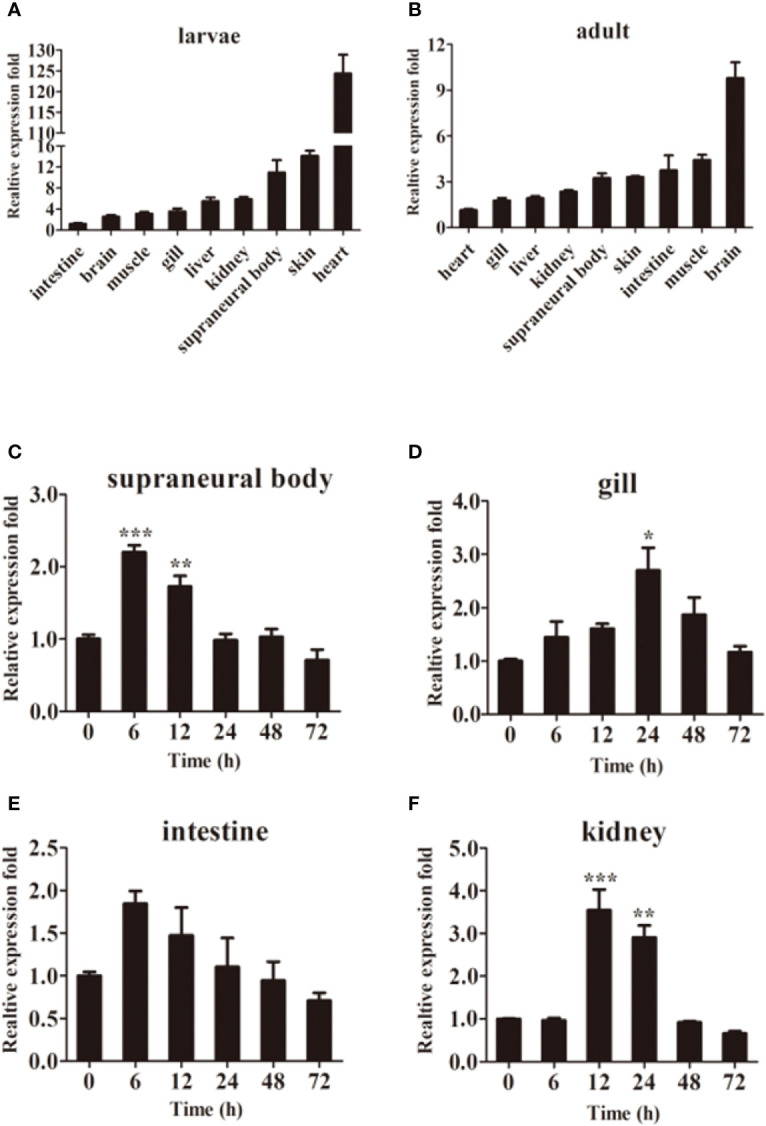
Tissue expression of LmTLR14d. Relative expression of *LmTLR14d* in different tissues of larvae **(A)** and adult **(B)**. *LmTLR14d* expression in adult SB **(C)**, gill **(D)**, intestine **(E)**, and kidney **(F)** challenged by *P. aeruginosa* at 0, 6, 12, 24, 48 and 72 h 2^-ΔΔCT^ method was used to calculate the relative expression level. β-actin was used as an internal control for qPCR. Bars show mean ± SEM. A one-way ANOVA analysis of variance followed by the Bonferroni multiple comparison test. * means significant difference (n=3, *P<0.05 **P<0.01 ***P<0.001).

The qPCR results showed that *LmTLR14d* differed in their expression profiles. *LmTLR14d* is highly expressed in the heart, SB, and skin of larvae, while it is highly expressed in the brain, intestine, skin, SB, and muscle of adults. (**Figures 3A, B**).

Exposure to *P. aeruginosa* induced expression of *LmTLR14d* in various immune tissues. The infection resulted in a change in tissue profiles of *LmTLR14d* expression. After 6 h of infection, the expression of *LmTLR14d* in SB and intestine increased significantly and reached the highest level, and gradually decreased and stabilized thereafter. After infection, the expression of *LmTLR14d* in gills continuously increased. At 24 h, the expression of *LmTLR14d* was significantly different from that at 0 h, and then the expression of *LmTLR14d* gradually decreased. After 12 h of infection, the *LmTLR14d* expression level in the kidney reached the highest level, then gradually decreased and stabilized (**Figures 3C–F**).

### Subcellular localization of LmTLR14d

**Figure 4:**
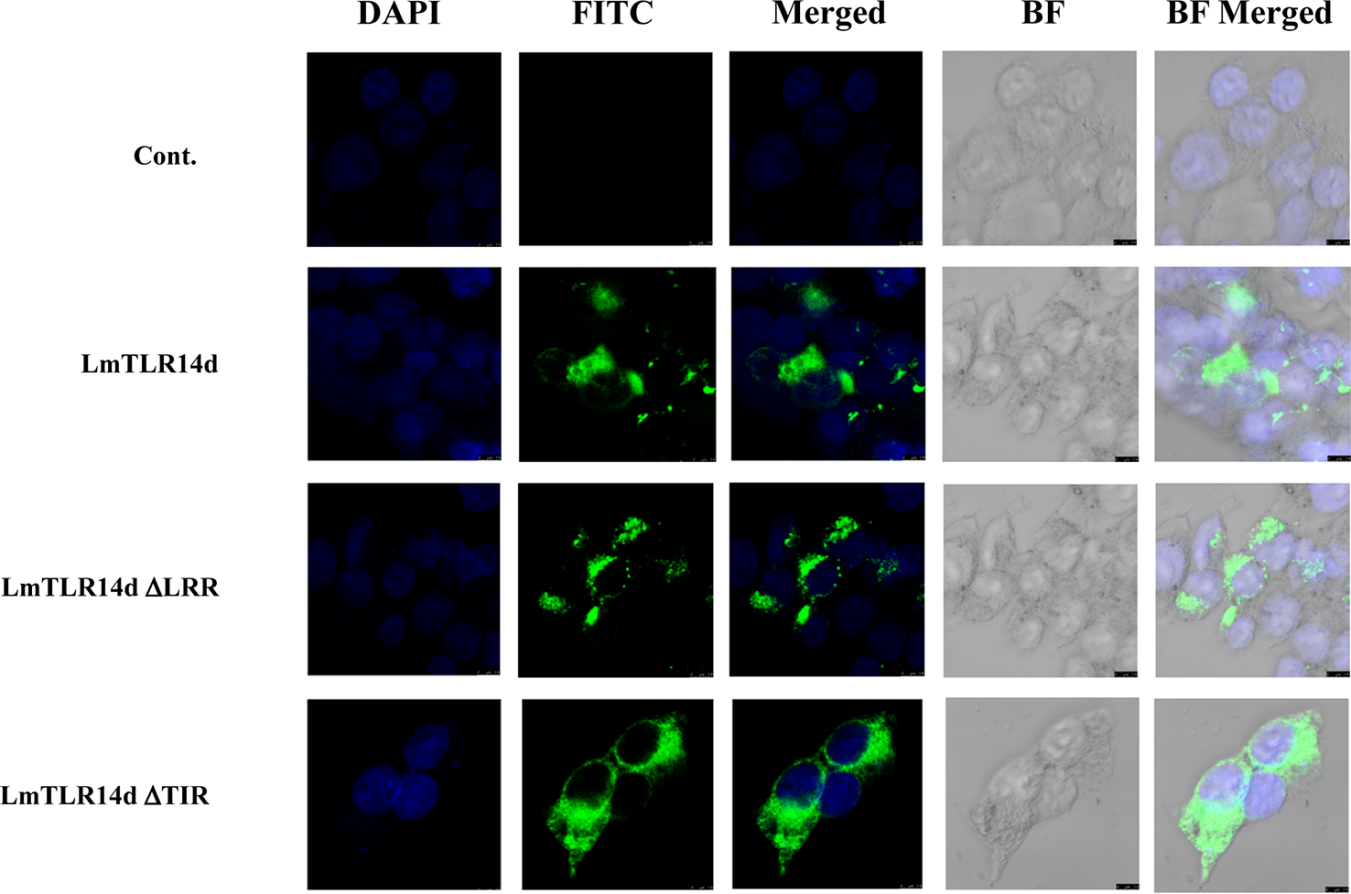
Localization of LmTLR14d, LmTLR14d ΔLRR and LmTLR14d ΔTIR in HEK 293T cells. DAPI stained nuclei (blue fluorescence, DAPI fluorescence channel), FLAG-tagged antibody labeled pCMV-LmTLR14d, pCMV-LmTLR14d ΔLRR and pCMV-LmTLR14d ΔTIR fusion protein (green fluorescence, FITC fluorescence channel), and bright field observations. Empty plasmid (pCMV) was used as a control, the scale is shown in the figure.

LmTLR14d was localized in the cytoplasm and distributed in clusters around the nucleus. Similarly, the truncated LmTLR14d protein lacking its LRR domain were localized in the cytoplasm as clusters. The truncated LmTLR14d protein lacking its TIR domain was uniformly distributed in the cytoplasm (**Figure 4**).

### Physical interaction between LmTLR14d and adaptor proteins

**Figure 5:**
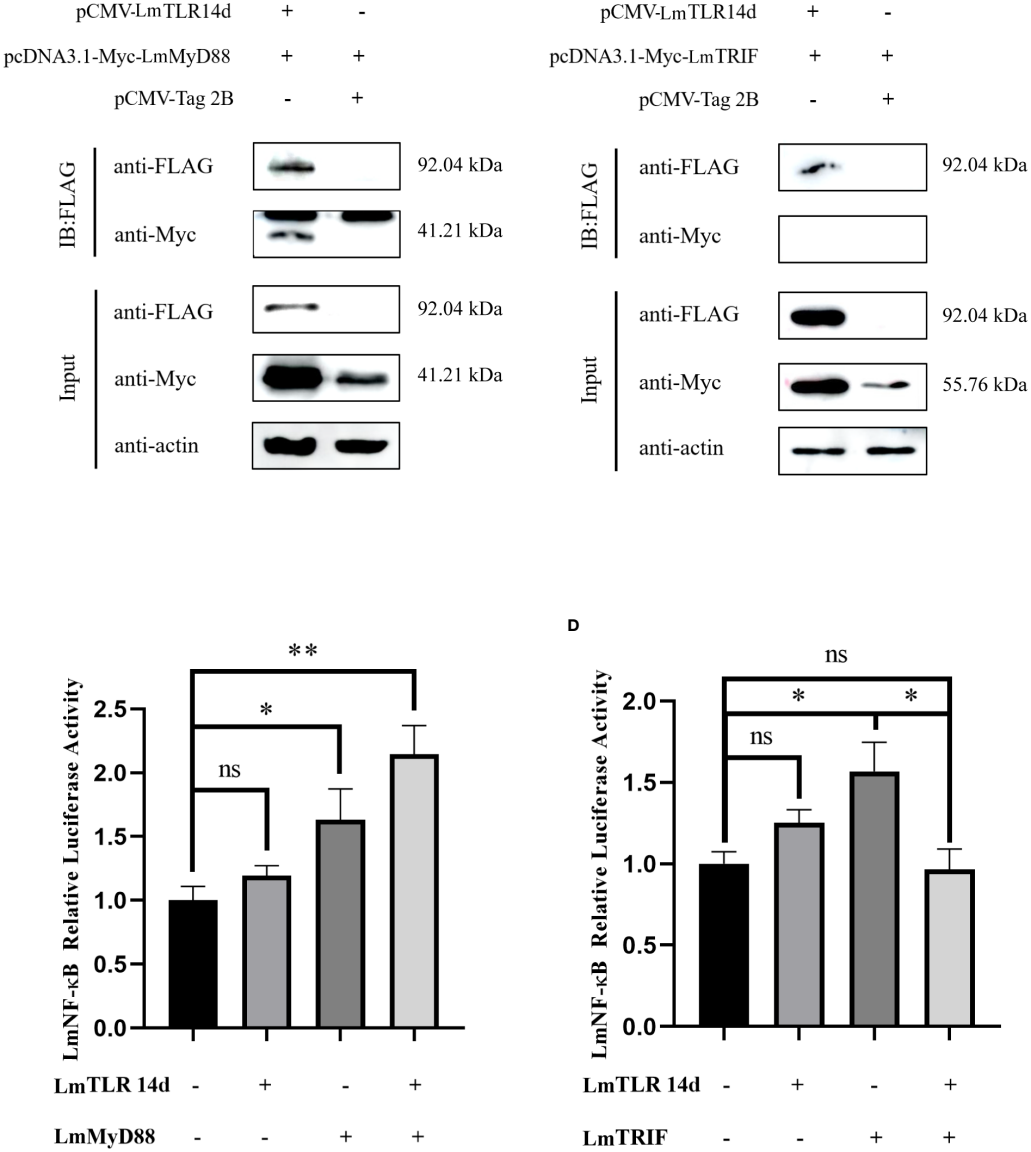
Relationship between LmTLR14d and TLR signal-related molecules in lampreys. The physical interaction between LmTLR14d and LmMyD88 **(A)**, LmTRIF **(B)** in HEK 293T cells. FLAG-LmTLR14d was co-transfected with Myc-LmMyD88 and Myc-LmTRIF, respectively. Immuno-coprecipitation was performed with protein A magnetic beads that bound anti-FLAG antibodies. *β-actin* as the internal reference. The samples before and after immunoprecipitation were separated by SDS-PAGE and Western blotting was performed with anti-FLAG or anti-Myc antibodies. **(C)** The effect of co-transfection of LmTLR14d and LmMyD88 on LmNF-kB. **(D)** The effect of co-transfection of LmTLR14d and LmTRIF on LmNF-κB. Bars show mean ± SEM. A one-way ANOVA analysis of variance followed by the Bonferroni multiple comparison test. * means significant difference (n=3, *P<0.05 **P<0.01). Ns stands for no significant difference.

After co-transfection, the fusion proteins corresponding to LmTLR14d, LmMyD88, and LmTRIF were all expressed in HEK 293T cells. The expression of LmTLR14d was detected by immunoprecipitation. LmTLR14d precipitated with LmMyD88, but not with LmTRIF (**Figures 5A, B**).

### Effects of LmTLR14d on LmNF-κB

LmTLR14d, LmMyD88, and LmTRIF enhanced the activity of the LmNF-κB promoter alone. LmTLR14d co-transfected with LmMyD88 significantly enhanced NF-κB activity, and compared with the LmMyD88 and LmTLR14d transfection groups alone, the NF-κB was promoted more significantly. However, the co-transfection of LmTLR14d and LmTRIF significantly reduced the promoting effect of NF-κB compared with LmTRIF alone transfection group, and the activity of NF-κB was lower than that of LmTLR14d and LmTRIF alone transfection group (**Figures 5C, D**).

### Effect of LmTLR14d on downstream inflammatory cytokines

**Figure 6:**
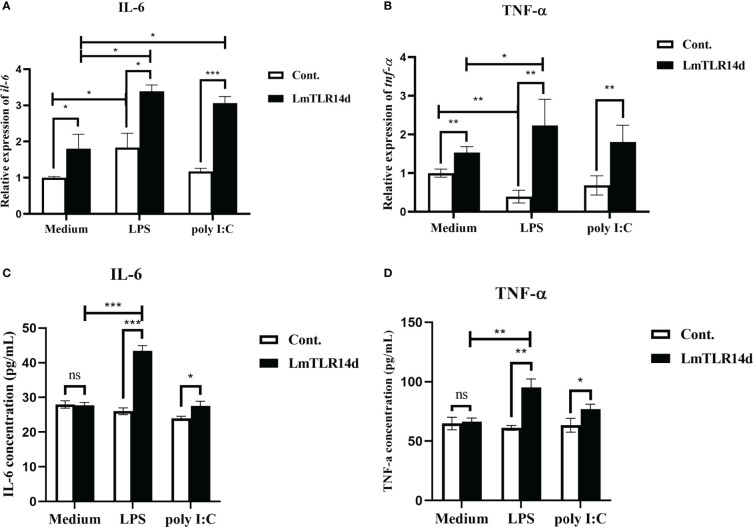
Effects of LmTLR14d on inflammatory cytokines downstream of NF-κB signal under natural state, LPS stimulation (12 h of incubation) and poly I:C stimulation (12 h of incubation). Quantitative real-time PCR (qPCR) showing effects of LmTLR14d overexpression on *il-6* **(A)** and *tnf-α* **(B)** in HEK 293T cells. ELISA showing LmTLR14d regulated the production of inflammatory cytokines IL-6 **(C)** and TNF-α **(D)** in HEK 293T cells. Bars show mean ± SEM. A one-way ANOVA analysis of variance followed by the Bonferroni multiple comparison test. * means significant difference (n=3, *P<0.05 **P<0.01 ***P<0.001). Ns stands for no significant difference.

The mRNA expression of inflammatory cytokines downstream of NF-κB signal showed that whether in natural state, after LPS or poly I:C stimulation, the overexpression of LmTLR14d can significantly increase the mRNA level of inflammatory cytokine genes *il-6* and *tnf-α*. On the premise of over-expression of LmTLR14d, the expression of *il-6* in LPS-stimulated group and poly I:C-stimulated group was significantly higher than that in natural state. Similarly, the expression of *tnf-α* in LPS-stimulated group was significantly higher than that in natural state, and the expression of *tnf-α* in poly I:C-stimulated group was higher than that in natural state, but not significantly (**Figures 6A, B**). Correspondingly, we detected the expression of these inflammatory cytokine proteins. The results of ELISA showed that overexpression of LmTLR14d can significantly promote the secretion of inflammatory cytokines IL-6 and TNF-α after LPS stimulation or poly I:C stimulation. On the premise of LmTLR14d overexpression, the production of IL-6 and TNF-α after LPS stimulation was significantly higher than that in natural state (**Figures 6C, D**).

## Discussion

There are four homologues of TLR14 in lamprey, including TLR14a, TLR14b, TLR14c and TLR14d. It is suggested that this molecule has more diverse and important function in the long-term evolutionary process. TLR14a and TLR14b in sea lamprey are homologous genes of TLR14a and TLR14b in Japanese lamprey (27). TLR14c and TLR14d in sea lamprey are paralogous genes of TLR14a and TLR14b in Japanese lamprey, which have the highest similarity with zebrafish TLR18 (28). However, although TLR14d was previously annotated in sea lamprey, the annotation did not cover the complete protein coding sequence of TLR14d. The domain analysis of sea lamprey TLR14d lacks the N-terminal region (28). In this study, *LmTLR14d* was identified as homologous genes of sea lamprey TLR14d. Moreover, according to the identification results, we found the LmTLR14d homologous gene annotated as PmTLR2 in the sea lamprey genome. LmTLR14 was composed of a classical structure, including extracellular, transmembrane, and intracellular regions. According to the genome sequence, an LRRCT domain, a transmembrane domain, and a TIR domain from the partial cds sequence of sea lamprey TLR14d have been annotated (28). After obtaining the complete cds sequence of LmTLR14d, we performed a detailed analysis of the LmTLR14d ECD. Lamprey TLR14d has a long N- terminal sequence before the LRR domain as compared to other vertebrate TLR1 subfamily members. However, this specific sequence does not have a classical domain for pathogen recognition (32). For the LRR domain of TLR, there is a conservative amino acid sequence arrangement of “LxxLxLxxNxL” or “LxxLxLxxCxxL”, where “L” is Leu, Ile, Val or Phe, “N” is Asn, Thr, Ser or Cys, and “C” is Cys, Ser or Asn (33). In addition, the protein structure of the LRR domain of TLR presents a single continuous structure, which is arc-shaped or horseshoe-shaped in conformation. Among them, the inner concave surface is a stack of parallel β stands, and the outer convex surface has multiple secondary structures such as α-helix and β-turns (34). Two cysteine clusters containing 2 to 5 cysteine residues are typically present flanking the continuous LRR domain. The N- terminal and C- terminal cysteine clusters of LRRs are referred to as LRRNT and LRRCT, respectively (35). Here, we annotated the existence of 1 LRRNT domain, 19 LRR domains, and 1 LRRCT domain in LmTLR14d through multi-sequence alignment. This result was verified by 3D homologous modeling. For the classification of TLRs, different TLR subfamilies have different numbers and motifs of LRR domains. Here, LmTLR14d has the characteristics of a classical TLR1 subfamily (33). The TIR structure contains three conservative box regions: box1 (Y/FHAFI/VSYSQ), box2 (LCVHERDFV/TPG), and box3 (FWAN/QLR). Studies have shown that LRR with a single continuous structure formed a horseshoe-shaped structure, and its concave surface was related to identifying various pathogens (36). The three conserved box domains in the TIR play an important role in TLRs signal transduction. Box1 and box2 participate in the downstream signal transduction process. Box2 forms an important loop structure, which has a highly conserved proline residue and cannot carry out signal transduction when the proline mutated to histidine. Box3 is closely related to the location of the receptor (37).

Based on phylogenetic tree analysis and sequence identity analysis, LmTLR14d belonged to the TLR1 subfamily. It is worth noting that TLR14 and TLR18 in fish are considered to be homologous molecules at present, so they are both involved in the following discussion (17, 18). LmTLR14d was clustered with TLR14/18 of teleost fish. TLR14 and TLR18 in teleost fish belong to the TLR1 subfamily (38). Interestingly, Japanese lamprey TLR14a/b and bony fish TLR25 are grouped. This is because when the Japanese lamprey TLR14a/b was identified, the bony fish TLR25 had not been identified and further homology analysis could not be done (27, 39). Except for homologous genes, LmTLR14d has the highest sequence identity with bony fish TLR14/18 protein, while Japanese lamprey TLR14a/b has the highest sequence identity with bony fish TLR25 protein. The homology of LmTLR14d with bony fish TLR14/18 is higher than that with LjTLR14a/b. Although LjTLR14a/b was initially classified as TLR14, its consistency comparison with bony fish TLR14 (Identity with tiger puffer TLR14: LjTLR14a:32%, LjTLR14b:34%) at that time was similar to that in this study (Identity with bony fish TLR14/18: LjTLR14a: 31.90-32.99%, LjTLR14b: 32.88-33.59%) (27). In addition, the protein sequence identity of the LmTLR14d intracellular segment is generally higher than that of the extracellular segment. Therefore, the intracellular segment of LmTLR14d is more conservative than the extracellular segment. These results showed that LmTLR14d had similar structural and phylogenetic positions with other bony fish TLR 14/18, which might have functions consistent with bony fish TLR14/18. The previously identified Japanese lamprey TLR14a/b is more like the homologous molecule of fish TLR25.

The expression pattern of *LmTLR14* revealed its constitutive and inducible characteristics. TLR14a and TLR14b showed the highest expression in the gill of sea lamprey (28). In teleosts, Japanese flounder TLR14 shows the highest expression in the head kidney (20). Teleosts TLR18 was highly expressed in the gill, kidney, or skin (18, 39, 40). These studies indicated that the expression profiles of TLR in different species were not identical, but it was usually relatively high in immune-related tissues. In this study, *LmTLR14d* was highly expressed in immune-related tissues such as the intestine, skin, and SB. In addition, we noticed that LmTLR14d was highly expressed in larval heart, but relatively low in adult heart. Larval stage is the key stage of heart and blood vessel development. Research shows that TLR signal is related to the development of fish larvae (41). That may be the reason why LmTLR14d is highly expressed in larval heart. Different expression patterns might be due to species and different developmental stages. The extensive *LmTLR14d* expression revealed that they maybe play an important role in the immune surveillance system of host tissues.

TLR genes are widely involved in the immune response to bacteria challenges in TLR signaling pathways. Up to date, many documents showed that TLR14 and TLR18 increased significantly when the host was challenged by bacteria or bacterial-derived TLR agonists. For example, the expression of TLR14 in the kidney of Japanese flounder was significantly increased after 6 days of infection with *Edwardsiella tarda* (20). The expression of TLR18 increased significantly in the spleen of common carp (*Crprinus carpio*) and grass carp (*Ctenopharyngodon idella*) after infection by *Aeromonas hydrophila* (18, 40). By contrast, the expression of *LmTLR14d* increased significantly in SB, gill, and kidney tissues when Northeast Chinese lamprey was challenged with *P. aeruginosa*. However, there was no significant change in the expression of LmTLR14d in the intestine, suggesting that it may be less involved in the immune response induced by bacteria in the intestine. These results indicated that the LmTLR14d can induce immune responses to bacteria in some immune tissues, but the mechanism at the mRNA level remains to be further studied.

The subcellular localization morphology of LmTLR14d is similar to that of fish-specific TLR14/18, and they are all clustered in the cytoplasm (42). It suggested that the specific TLR14/18 was evolutionarily conservative. From the truncated LmTLR14d protein subcellular localization result, it is suggested that the subcellular localization of LmTLR14d was determined by its TIR domain. The subcellular localization of some intracellular TLRs is also determined by the TIR domain (43). Our result is consistent with the localization of TLR1 found in miiuy croaker (44).

Since our previous experiments have shown that LmTLR14d is structurally conserved and has an important function in the innate immune response against bacterial infection, we questioned which adaptor-dependent pathway does LmTLR14d mediate immune response.MyD88 and TRIF are known to be important adaptor proteins in the TLR-mediated immune signaling pathway (45). Our co-precipitation results showed that LmTLR14d could recruit adaptor LmMyD88, but could not recruit adaptor LmTRIF. MyD88 is an adapter molecule of the classical TLR1 subfamily (46). MyD88 is also an adapter molecule of fish-specific TLR14/18. Interestingly, Asian swamp eel TLR14 cannot recruit TRIF, but Nile tilapia (*Oreochromis niloticus*) TLR18 can recruit TRIF (24, 42). The ability of fish-specific TLR14/18 to recruit TRIF is not conservative. NF-κB is an important transcriptional regulator and widely present in cells. Previous studies have shown that NF-κB was widely involved in TLR-mediated immune signaling pathways (47). Both LmMyD88 and LmTRIF could significantly promote LmNF-κB activity (30, 31). Actually, in the classical TLR signal pathway, the adaptor of TLR, both MyD88 and TRIF can induce the production of inflammatory cytokines and active innate immune response by promoting NF-κB downstream of TLR signal (48, 49). We found that each of LmTLR14d, LmMyD88, and LmTRIF can independently promote LmNF-κB activity, and the co-overexpression of LmTLR14d and LmMyD88 significantly promoted LmNF-κB activity. In contrast, the co-overexpression of LmTLR14d and LmTRIF significantly reduced the promotion of LmNF-κB. The classical TLR1 subfamily members mainly induce NF-κB to enter the nucleus and bind to MyD88. Although there are differences in homology with lamprey TLR14d, LjTLR14b can also activate NF-κB through MyD88 (27). Similar to results of this study, overexpression of Asian swamp eel TLR14 promoted NF-κB activity (24). In teleosts, the co-overexpression of Miiuy croaker TLR1 and MyD88 promotes the activity of NF-κB, and the inhibition of TLR1 reduces the promotion (44). In addition, Nile tilapia TLR18 (OnTLR18) could bind OnMyD88, and OnTLR18 could activate NF-κB, suggesting that teleosts TLR18 might regulate downstream immune transcription factors by binding MyD88 (42). Our co-transfection results of LmTLR14 and LmTRIF further confirmed the phenomenon of immune coprecipitation. TRIF is only involved in the signaling of TLR3 and TLR4, however, all TLRs except for TLR3, can signal through the adaptor molecule MyD88. LmTLR14d belongs to the TLR1 subfamily with conserved structure and function, so we suggest that it cannot activate NF-κB signaling through the TRIF adaptor. Our results suggested that as a homologous gene of bony fish TLR14/18, LmTLR14d may activate NF-κB by recruiting LmMyD88.

Interleukin 6 (IL-6) and tumor necrosis factor-α (TNF-α) are inflammatory cytokines regulated by NF-κB (50). In order to further explore the regulation of LmTLR14d on NF-κB signal, we detected the effect of LmTLR14d on inflammatory cytokines downstream of NF-κB signal *in vitro*. In the study of fish TLR signal, zebrafish TLR can activate NF-κB to induce the production of downstream inflammatory cytokine IL-6 (51). We found that LmTLR14d can induce the expression of *il-6* and *tnf-α in vitro*. Similarly, Asian swamp eel TLR14 can induce the expression of downstream inflammatory cytokines *il-6* and *tnf-α in vitro* (24). In mammals, both immunostimulants LPS and poly I:C can induce the activation of NF-κB (52). We found the induction of inflammatory cytokines IL-6 and TNF-α by LmTLR14d was further enhanced after LPS stimulation. LmTLR14d may activate NF-κB and promote the secretion of inflammatory cytokines after recognizing the derivatives of Gram-negative bacteria. In fact, both golden pompano and mandarin fish TLR14 can be induced by LPS (22, 23), suggesting that fish TLR14 can recognize Gram-negative bacteria to activate the downstream immune response. In the classical signal pathway, poly I:C can induce the production of inflammatory cytokines such as IL-6 (53). After stimulation by poly I:C, the induced expression of inflammatory cytokine gene *il-6* in the HEK293T cells with overexpressed LmTLR14d, was further enhanced in protein and mRNA level. Mammalian TLR3 can mediate the activation of NF-κB after recognizing poly I:C (54), and our results suggest that LmTLR14d may have a similar recognition mediation function.

## Summary

In this study, we report the structure and function of jawless vertebrates (cyclostomes) TLR14d gene. Northeast Chinese lamprey TLR14d has one LRRNT domain, 19 LRR domains, one LRRCT domain, and one TIR domain, which is a homologous gene of bony fish TLR14/18. *LmTLR14d* was expressed in all tissues tested, and the expression level changed significantly when the lamprey was challenged by *P. aeruginosa*. LmTLR14d is located in the cytoplasm in clusters, and its subcellular localization is determined by the TIR domain. LmTLR14d can recruit LmMyD88, and activate the transcription factor NF-κB, but LmTRIF does not have this function. LmTLR14d can further induce the expression of inflammatory cytokine genes *il-6* and *tnf-α* downstream of NF-kB signal both in protein and mRNA level. These results indicate LmTLR14d is highly conserved with jawed fish TLR14, and, as the ancestor of jawed fish TLR14, it participates in the innate immune response by initiating the downstream NF-κB pathway through MyD88 adaptor molecule. This study further increases our understanding of the function and origin of fish-specific TLRs.

## Data availability statement

The original contributions presented in the study are included in the article/**Supplementary Material**. Further inquiries can be directed to the corresponding author.

## Ethics statement

The animal study was reviewed and approved by the ethics committee of laboratory animals of Shanghai Ocean University.

## Author contributions

ZZ: Formal analysis, Data curation, performed the experiments, analyzed the data, Writing-original draft. SD: Data curation, performed the experiments, analyzed the data. YW: Assisted in the completion of part of the experiment. JR: Formal analysis, Investigation. XZ: Formal analysis, Investigation. WL: Conceptualization and editing. QZ: Conceived, designed the experiments, writing-review & editing. All authors contributed to the article and approved the submitted version.
