# Supplementary material for: Identification and characterization of Toll-like receptor 14d in Northeast Chinese lamprey (Lethenteron morii)
Source: Front Immunol. 2023 Apr 18;14:1153628. doi: 10.3389/fimmu.2023.1153628 (PMC10151648; doi:10.3389/fimmu.2023.1153628)
Supplement: Supplementary file 1 [file DataSheet_1.docx]

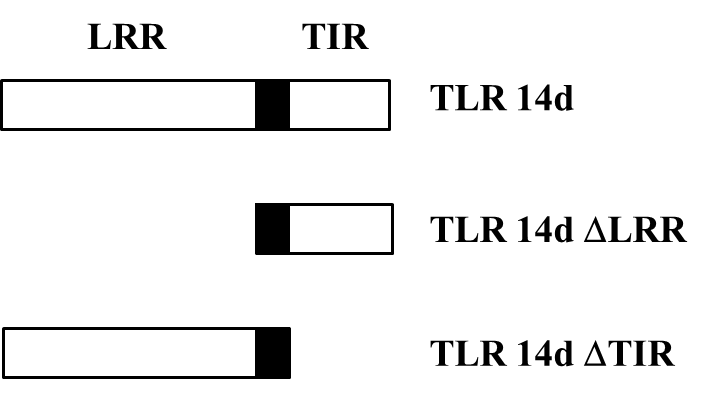


**SFig. 1** Construction fragment of *in vitro* overexpression plasmid of LmTLR14d.


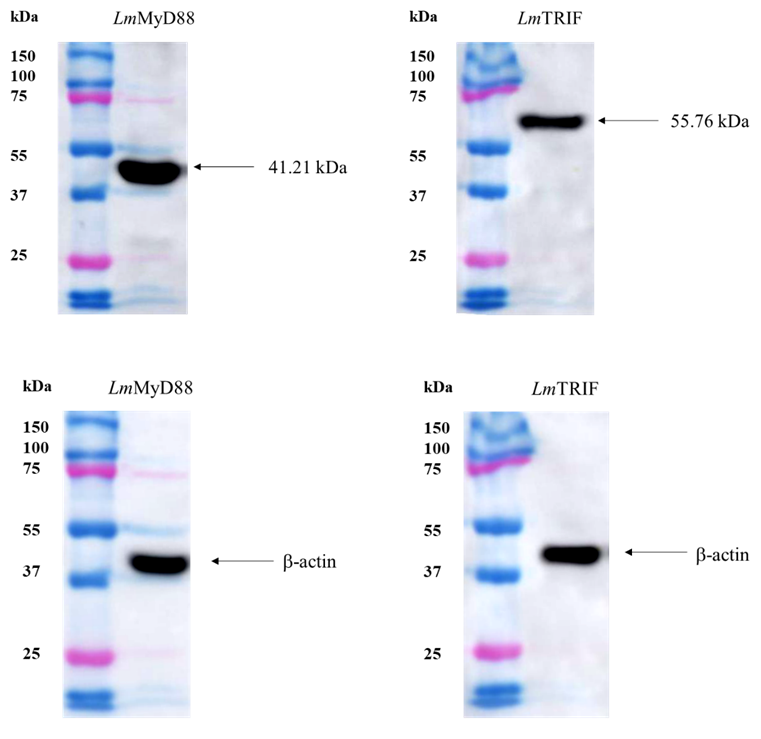


**SFig.2** Expression of pcDNA3.1(+)-Myc-LmMyD88 and pcDNA3.1(+)-Myc-LmTRIF in HEK 293T cells.
